# Water Extract of *Inula japonica* Flower Ameliorates *Dermatophagoides farinae* Extract-Induced Atopic Dermatitis-like Skin Inflammation by Attenuating JAK/STAT Signaling

**DOI:** 10.3390/ijms26157063

**Published:** 2025-07-22

**Authors:** Ki-Shuk Shim, Hye Jin Kim, Dong Ryun Gu, Seong Cheol Kim, Ik Soo Lee, Sung-Wook Chae, Musun Park, Taesoo Kim, Ki Mo Kim

**Affiliations:** 1 KM Convergence Research Division, Korea Institute of Oriental Medicine, Yuseong-daero 1672, Yuseong-gu, Daejeon 34054, Republic of Korea; angeloshim@kiom.re.kr (K.-S.S.); kimhyejin43@kiom.re.kr (H.J.K.); mrwonsin@kiom.re.kr (D.R.G.); knifer48@kiom.re.kr (I.S.L.); kendall@kiom.re.kr (S.-W.C.); 2 Biohealthcare R & D Center, Hynundai Bioland Co., Ltd., Manhae-ro 152, Danwon-gu, Ansan 15407, Republic of Korea; iron0907@hyundaibioland.co.kr; 3 KM Data Division, Korea Institute of Oriental Medicine, Yuseong-daero 1672, Yuseong-gu, Daejeon 34054, Republic of Korea; bmusun@kiom.re.kr

**Keywords:** *Inula japonica*, atopic dermatitis, skin inflammation, JAK/STAT

## Abstract

The *Inula japonica* flower is traditionally used to alleviate lung inflammatory symptoms. While the therapeutic effect of the *I. japonica* flower on lung diseases has been suggested, the efficacy of the *I. japonica* flower in treating atopic dermatitis (AD) remains unknown. We investigated the effects of a water extract of the *I. japonica* flower (WEIF) on *Dermatophagoides farinae* extract (DfE)-induced AD-like inflammation in NC/Nga mice. Histological analysis of the epidermal structure, mast cell infiltration, and barrier protein expression were examined. Serum inflammatory mediator levels were assessed. To elucidate the regulatory pathway of WEIF, the effects of 1,5-dicaffeoylquinic acid (DCQA) and 1-O-acetylbritannilactone (ABL) in WEIF on the JAK/STAT pathway were evaluated in interferon-γ/tumor necrosis factor (TNF)-α-stimulated human adult epidermal keratinocytes. WEIF ameliorated DfE-induced skin inflammation by reducing dermatitis scores, mast cell infiltration, skin structural damage, and serum inflammatory mediator levels. Additionally, DCQA and ABL significantly inhibited JAK/STAT activation in interferon-γ/TNF-α-treated keratinocytes. Furthermore, ligand-binding analysis revealed high binding affinities of DCQA and ABL for JAK. These results suggest the pharmacological potential of WEIF to alleviate DfE-induced skin inflammation by inhibiting the JAK/STAT signaling pathway. In conclusion, these findings support the development of WEIF as a therapeutic treatment for AD-like skin inflammatory diseases.

## 1. Introduction

Atopic dermatitis (AD) is a prevalent inflammatory skin disease that is common in young children, and its incidence is often associated with increased exposure to allergen-rich environments [1]. While commonly manifesting in childhood, AD frequently presents latent onset or resurgence in adulthood, making therapeutic approaches challenging and resulting in a chronic skin disease [2,3]. AD pathogenesis is characterized by skin barrier dysfunction coupled with immune dysregulation, including hyperactivity of skin-resident cells, such as keratinocytes, and an imbalance in Th immunity [4,5]. Allergens increase keratinocyte differentiation, which further exacerbates skin barrier dysfunction and leads to a reduction in the water content of the skin [4,6]. Allergens also stimulate keratinocytes and resident skin immune cells to produce proinflammatory mediators, including cytokines and chemokines. These mediators induce T-cell stimulation and Th1/2 imbalance, favoring the Th2-dominant immune response in the acute stage of AD [5,7]. Proinflammatory cytokines, such as interferon (IFN)-γ and tumor necrosis factor (TNF)-α, also stimulate signal transducer and activator of transcription (STAT) signaling in keratinocytes to produce various inflammatory chemokines and cytokines [8,9], which recruit immune cells into the skin lesion. Th2 inflammatory cytokines, such as interleukin (IL)-4, reduces the expression of skin barrier proteins by activating the Janus kinase (JAK)/STAT pathways in keratinocytes [10]. However, JAK inhibition restores skin barrier protein expression and ameliorates AD-like skin inflammation [11,12].

The topical application of corticosteroids, such as dexamethasone, has been commonly employed as a first-line treatment for the clinical management of AD to relieve excess skin inflammation [13]. However, the reduced efficacy of long-term use and/or several comorbidities, such as skin atrophy, severe acne, or adrenal suppression, have been suggested as drawback in the clinic [14]. Chemical or biological candidates that regulate the JAK/STAT pathway have received much attention for the development of clinical or pharmaceutical treatments for AD-like skin inflammatory diseases [15]. Thus, researchers have actively explored the development of novel therapeutic options derived from natural herbs or their active components that have the potential to suppress AD-like skin inflammation by modulating JAK/STAT signaling [8,16], providing promising alternatives therapeutics with potentially fewer adverse effects.

Nishiki-nezumi Cinnamon/Nagoya (NC/Nga) mice are inbred strains that naturally exhibit AD-like skin characteristics under conventional housing conditions [17]. Skin sensitization to various allergens, such as house dust mites, in NC/Nga mice induces hypersensitivity to the immune response and immunological imbalance and exacerbates skin barrier dysfunction, leading to the development of AD symptoms [18,19]. *Dermatophagoides farinae* (DfE), a major household allergen related to AD, commonly resides in bedding and is frequently transferred between clothing in daily life [20]. The immunological and histological features of DfE-sensitized NC/Nga mice closely parallel those symptoms observed in AD patients [21], suggesting that this model is a valuable in vivo tool for evaluating the pharmaceutical efficacy of therapeutic agents in the treatment of skin inflammatory diseases, such as AD.

*Inula japonica* Thunb., classified in the Asteraceae family, has been traditionally used to alleviate symptoms of lung inflammation, such as phlegm, cough, and bronchitis [22]. Consistent with these traditional uses, recent studies have reported the pharmacological activities of *I. japonica* or its constituents against lung inflammation. The *I. japonica* flower and its constituent, britanin, inhibit ovalbumin (OVA)-induced airway inflammation in murine asthma models by reducing eosinophil recruitment and lowering Th2 cytokine and immunoglobulin E (IgE) levels [23]. Additionally, 1β-hydroxy alantolactone, the main sesquiterpene lactone from *I. japonica*, alleviates bleomycin-induced pulmonary fibrosis in the lungs of rats by inhibiting the c-Jun N-terminal kinase (JNK)/NF-κB pathway [24]. Furthermore, 1β-hydroxy alantolactone suppresses 2,4-dinitrochlorobenzene-induced AD-like skin lesions in BALB/c mice by reducing the serum levels of proinflammatory cytokines, such as IL-1 [25], highlighting the anti-inflammatory potential of *I. japonica* in the context of AD-like skin inflammation. Traditional medicinal candidates, which have therapeutic effects on asthma, have exhibited beneficial effects on other inflammatory diseases, such as AD and allergic rhinitis [26,27]. The water extract of the *I. japonica* flower (WEIF) ameliorates airway inflammation induced by OVA by inhibiting Th2 immunity through JAK/STAT signaling [23]. However, the pharmacological effect of WEIF on AD-like skin inflammation remains unknown. Because DfE is the most prevalent allergen that causes AD in daily life, the effect of WEIF on model mice with DfE-induced AD-like skin lesions needs to be evaluated. Therefore, this study aimed to explore the effects of WEIF using a DfE-induced AD-like skin inflammation model. As we previously identified 1,5-dicaffeoylquinic acid (DCQA) and 1-O-acetylbritannilactone (ABL) for main components of WEIF using UPLC-TQ-MS/MS analysis [23], the mechanisms of action of these components of WEIF were explored on the JAK/STAT signaling pathway in IFN-γ/TNF-α-stimulated normal human adult epidermal keratinocytes (HEKs). Additionally, we analyzed the molecular binding affinities of these components for JAK1/2 and STAT3.

## 2. Results

### 2.1. WEIF Ameliorates DfE-Induced AD-like Skin Lesion and Reduces Their Severity

**Figure 1:**
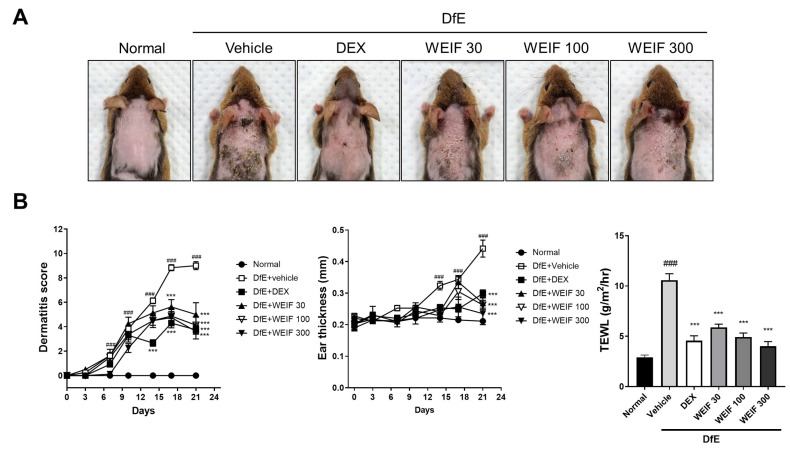
Effect of the water extract of *I. japonica* flower (WEIF) on *Dermatophagoides farinae* extract (DfE)-induced atopic dermatitis (AD)-like skin inflammation in NC/Nga mice. (**A**) Representative image of the inflamed dorsal skin in Nishiki-nezumi Cinnamon/Nagoya (NC/Nga) mice. (**B**) Time-course analysis of dermatitis scores and ear thicknesses of AD model mice over the experimental timeline. Transepidermal water loss (TEWL) values were measured on the final day of the experiment. The mice were sensitized to 4% sodium dodecyl sulfate (SDS) and subsequently challenged with DfE ointment (100 mg) twice weekly for 3 weeks to induce AD-like skin inflammation. The mouse group included the normal (control, ●), AD (DfE-induced + vehicle, □), AD + DEX (DfE-induced + dexamethasone 1 mg/kg, ■), AD + WEIF 30 (DfE-induced + WEIF 30 mg/kg, ▲), AD + WEIF 100 (DfE-induced + WEIF 100 mg/kg, ▽), and AD + WEIF 300 (DfE-induced + WEIF 300 mg/kg, ▼) groups. The data are presented as the means ± standard errors of the means (SEMs) (*n* = 6). The significance was set as ### *p* < 0.001 vs. the normal group; *** *p* < 0.001 vs. the AD group.

We established an AD-like skin inflammation mouse model via sodium dodecyl sulfate (SDS) sensitization followed by repeated topical application of DfE to the dorsal skin of mice [9]. DfE induction resulted in the development of AD-like skin lesions characterized by severe pathological features, including dryness, pruritus, papules, and lichenification (Figure 1A). WEIF (30–300 mg/kg) significantly ameliorated these pathological symptoms in a dose-dependent manner, albeit less effectively than dexamethasone did, in AD model mice. Starting from day 18, all three WEIF-treated groups presented significant reductions in dermatitis scores, which persisted until the end of the experiment, similar to the efficacy observed in the dexamethasone-treated group (Figure 1B). The significant inhibitory effect of WEIF on the DfE-induced increase in ear thickness was evident in AD model mice on the final day of the experiment. Furthermore, while the AD group displayed a significant increase in transepidermal water loss (TEWL) values, which represent skin barrier disruption, all of the WEIF-treated groups presented significantly reduced TEWL values, which was consistent with the observed reductions in skin lesion severity. These findings collectively suggest that WEIF administration effectively alleviates DfE-induced AD-like skin inflammatory symptoms in vivo. The administration of three different doses of WEIF did not change the body weight or spleen weight compared with those of the normal group, suggesting that WEIF does not cause toxicity or adverse immune responses at the concentrations tested.

### 2.2. WEIF Ameliorates Skin Structure Damage in DfE-Induced AD Model Mice

**Figure 2:**
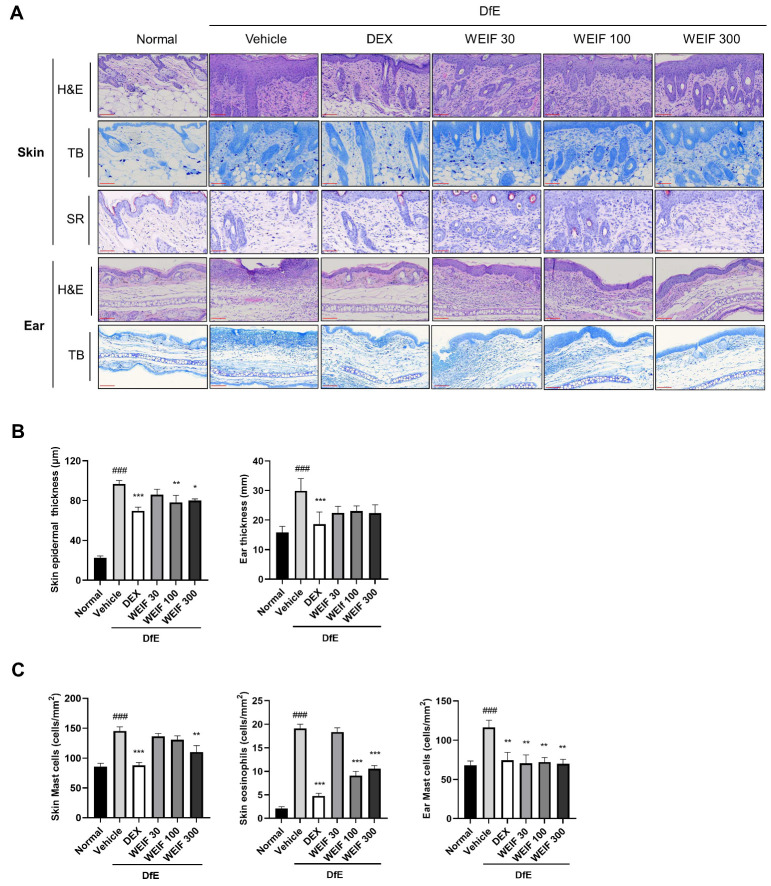
WEIF affects histological skin alterations in DfE-induced AD-like inflammation in NC/Nga mice. (**A**) Histological features of dorsal skin and ear sections stained with hematoxylin and eosin (H&E), toluidine blue (TB), and Sirius Red (SR). (**B**) Measurement of epidermal thickness in skin and ear sections. (**C**) Quantification of mast cell or eosinophil infiltration by counting the cells within randomly selected stained tissue areas at 200× magnification. The data are presented as a bar graph with the means ± SEMs (*n* = 6). The significance was set as ### *p* < 0.001 vs. the normal group; * *p* < 0.05, ** *p* < 0.01, *** *p* < 0.001 vs. the AD group. Scale bar (red color) = 100 μm.

AD skin lesions exhibit pathological characteristics, including hyperkeratosis and hyperplasia. In the context of the early response of AD inflammation, an increase in inflammatory factors stimulates keratinocyte differentiation, leading to hyperkeratosis and increased epidermal thickness, eventually resulting in the chronic phase of skin inflammation [28,29]. To elucidate the beneficial effects of WEIF on skin structural damage caused by DfE-induced skin inflammation, histopathological analysis of skin structure at the end of the animal experiment was conducted using hematoxylin and eosin (H&E) and toluidine blue (TB) staining. DfE application significantly increased the epidermal thickness and skin layer disruption in both the dorsal skin and ear tissues (Figure 2A,B). Conversely, WEIF administration significantly and dose-dependently reduced skin epidermal thickness, similar to the effects observed in the dexamethasone-treated group, suggesting a notable effect of WEIF on epidermal structure. WEIF administration tended to decrease ear tissue thickness, although the difference did not reach statistical significance (Figure 2B).

To assess inflammatory cell infiltration within the skin lesions, the number of mast cells in the dorsal skin and ear tissue sections was determined using TB staining. The number of eosinophils was examined using Sirius Red (SR) staining (Figure 2B). DfE application significantly increased mast cell and eosinophil accumulation in the dermal layer of the skin. However, WEIF markedly reduced the number of mast cells in both the dorsal skin and ear tissues, and the number of eosinophils in the dorsal skin tissues of AD model mice (Figure 2C). Collectively, these findings demonstrate that WEIF effectively mitigates the skin structural damage observed in DfE-induced AD-like skin inflammation model mice.

### 2.3. WEIF Protects Against DfE-Induced Downregulation of Skin Structural Proteins

**Figure 3:**
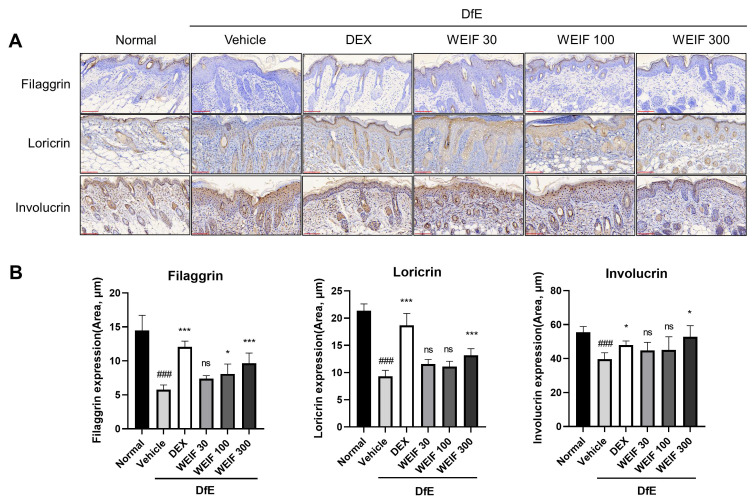
WEIF affects filaggrin and loricrin expression in the skin lesions of DfE-induced NC/Nga mice. (**A**) Immunohistochemical staining of filaggrin, loricrin, and involucrin expression in the dorsal skin lesions of the mice. The stained sections were examined under a microscope at 400× magnification. (**B**) Quantification of filaggrin, loricrin, and involucrin protein expression. The data are presented as bar graphs with the means ± SEMs (*n* = 6). ### *p* < 0.001 vs. the normal group; * *p* < 0.05, *** *p* < 0.001 vs. the AD group; ns, not significance. Scale bar (red color) = 100 μm.

WEIF ameliorated DfE-induced destruction of the skin barrier and restored physiological skin function, as demonstrated by the restored epidermal structures and decreased TEWL values in the model mice. To establish the mechanism by which WEIF alleviates barrier disruption, we analyzed the expression of the key skin structural proteins filaggrin, loricrin, and involucrin, which are critical for skin barrier integrity and hydration. DfE exposure significantly damaged the epidermal structure and substantially decreased the levels of these proteins (Figure 3A). Specifically, the expression of these proteins in the DfE-treated AD group relative to the normal group was three-fold lower for filaggrin, two-fold lower for loricrin, and a quarter-fold lower for involucrin (Figure 3B). Dexamethasone treatment nearly restored these protein levels to those observed in the normal group. Notably, WEIF (300 mg/kg) significantly attenuated the DfE-induced downregulation of skin barrier proteins, thereby restoring epidermal structure in AD-like skin inflammation model mice.

### 2.4. WEIF Attenuated the DfE-Induced Increase in Inflammatory Factor Levels

**Figure 4:**
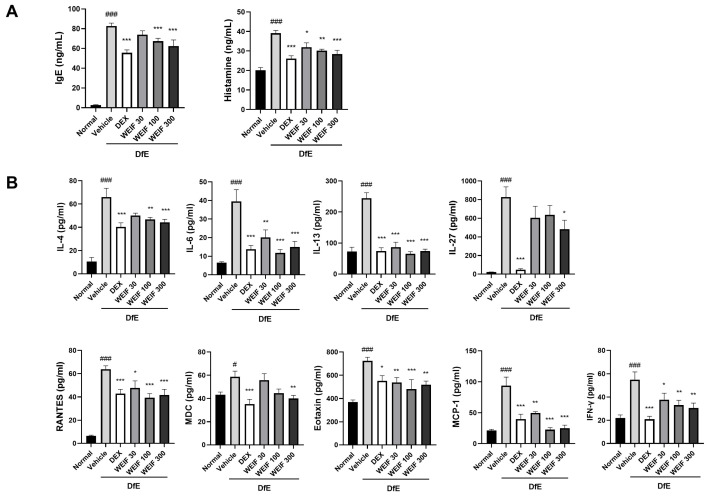
WEIF abrogated the DfE-induced changes in the serum levels of immunoglobulin E (IgE), histamine, and inflammatory factors in NC/Nga mice. (**A**) Serum IgE and histamine levels were determined using a sandwich ELISA. (**B**) Serum levels of nine inflammatory factors (IL-4, IL-6, IL-13, IL-27, RANTES, MDC, eotaxin, MCP-1, and IFN-γ,) were quantified using a bead-based immunoassay. The data are presented as bar graphs with the means ± SEMs (*n* = 6). # *p* < 0.05, ### *p* < 0.001 vs. the normal group; * *p* < 0.05, ** *p* < 0.01, *** *p* < 0.001 vs. the AD group.

Mast cell infiltration is a primary immune response to elevated serum IgE and histamine levels that induces mast cell granulation during skin inflammation. DfE challenge significantly increased the serum IgE and histamine levels, which were significantly attenuated by both dexamethasone and WEIF treatment (Figure 4A). Importantly, WEIF dose-dependently reduced both IgE and histamine levels, which is consistent with our observed phenotypical and historiological results. Infiltrated mast cells, eosinophils, or skin-resident cells, such as keratinocytes, generate T-cell stimulatory cytokines or chemokines in DfE-induced skin inflammation. Chronic AD is characterized by the predominant level of Th2 cytokines with a significant increase in Th1 cytokines. To elucidate the effects of WEIF on the generation of inflammatory mediators underlying this process, we analyzed the Th1-associated factors (regulated on activation, normal T-cell expressed and secreted (RANTES), IFN-α, and IL-27), Th2-associated factors (IL-4, IL-13, eotaxin and macrophage-derived chemokines (MDCs)), and pleiotropic factors (monocyte chemoattractant protein (MCP)-1 and IL-6) (Figure 4B). DfE exposure significantly increased the levels of these inflammatory mediators, which were effectively decreased by WEIF administration in a dose-dependent manner. The high dose of WEIF (300 mg/kg) had an inhibitory effect on these mediators comparable to that of the positive control, dexamethasone, with the exception of IL-27. These results underscore that WEIF suppresses the DfE-induced generation of multiple inflammatory mediators in an in vivo model, culminating in AD-like skin inflammation inhibition.

### 2.5. WEIF Components Suppress JAK/STAT Signaling

**Figure 5:**
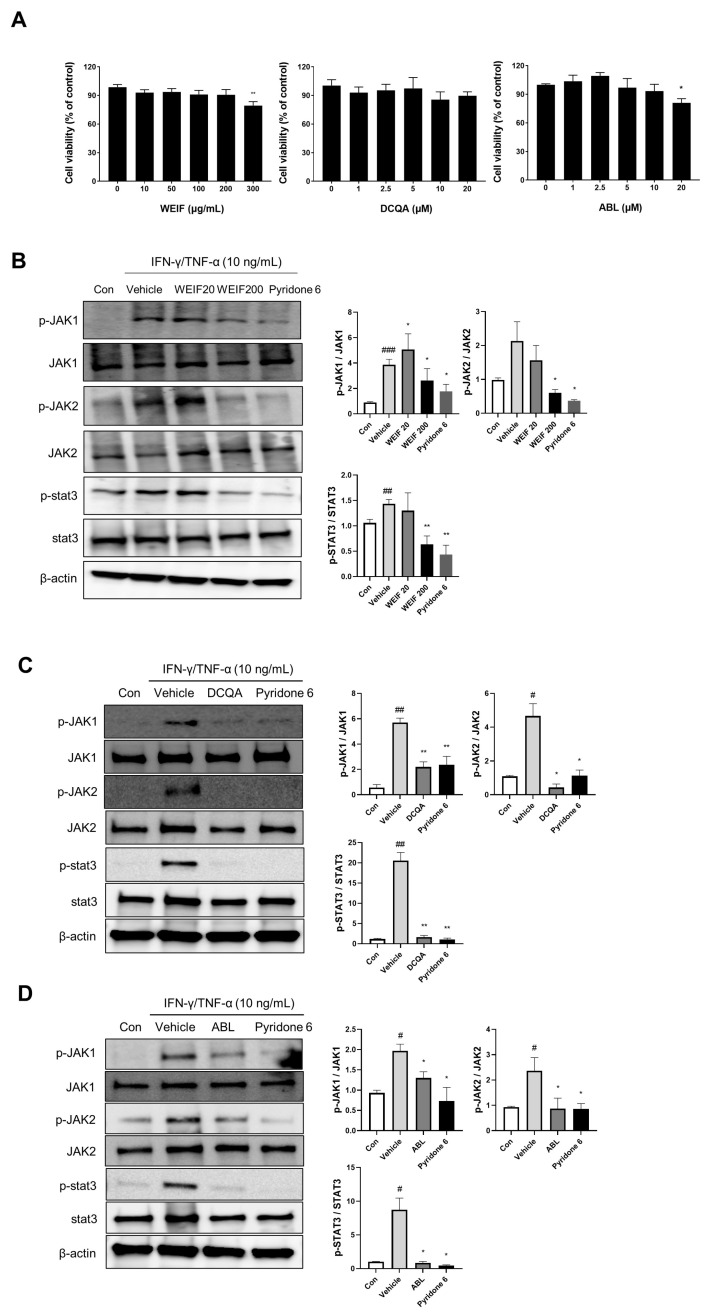
Effects of WEIF, 1,5-dicaffeoylquinic acid (DCQA), and 1-O-acetylbritannilactone (ABL) on JAK1/2 and STAT-3 phosphorylation in keratinocytes. (**A**) Cell viability was examined following 24 h of incubation with each sample. After pretreatment with (**B**) WEIF (20 or 200 µg/mL), (**C**) DCQA (10 µg/mL), or (**D**) ABL (10 µg/mL) for 1 h, the cells were stimulated with interferon (IFN)-γ/tumor necrosis factor (TNF)-α (10 ng/mL). Pyridone 6 was 10 nM. Total protein was extracted from the cells to examine Janus kinase (JAK)1/2 and signal transducer and activator of transcription (STAT)-3 phosphorylation by Western blot analysis. The intensities of the chemiluminescent signals are represented as a bar graph with the means ± SEMs from three independent experiments. # *p* < 0.05, ## *p* < 0.01, and ### *p* < 0.001 vs. the control; * *p* < 0.05 and ** *p* < 0.01 vs. the vehicle.

To investigate the pharmaceutical constituents of WEIF, we previously identified and quantified nine WEIF compounds using UPLC-TQ-MS/MS analysis [23]. DCQA and ABL were selected for this study because of their relatively high concentrations within WEIF and their structural similarity to other WEIF components. To elucidate the molecular mechanisms by which DCQA and ABL contribute to the anti-inflammatory effects of WEIF in a human cell model, we stimulated normal HEKs with IFN-γ and TNF-α (10 ng/mL). The HaCaT cells were pre-exposed to WEIF (0 to 300 µg/mL) and HEKs were treated with DCQA or ABL (0 to 20 µg/mL) for 24 h (Figure 5A). A total of 300 µg/mL of WEIF and 20 µg/mL of ABL significantly affected 20% of the cells, suggesting a maximum concentration of samples for subsequent cellular studies. Notably, IFN-γ/TNF-α induced increased phosphorylation of JAK1, JAK2, and their downstream effector, STAT3 (Figure 5B,C). However, WEIF (200 µg/mL) significantly attenuated IFN-γ/TNF-α-induced JAK1 and JAK2 phosphorylation, achieving a similar level of inhibition as the JAK inhibitor pyridone 6 (Figure 5B). DCQA and ABL (10 µg/mL) also significantly inhibited JAK1 and JAK2 phosphorylation, although to a lesser extent than pyridone 6 did (Figure 5C,D). Importantly, WEIF, DCQA, and ABL markedly abrogated STAT3 phosphorylation in keratinocytes (Figure 5B–D). These results suggest that the WEIF components DCQA and ABL contribute to the anti-inflammatory activity of WEIF by suppressing the JAK/STAT signaling pathway.

### 2.6. Docking Analysis of the WEIF Components with JAK1/2 and STAT3

**Figure 6:**
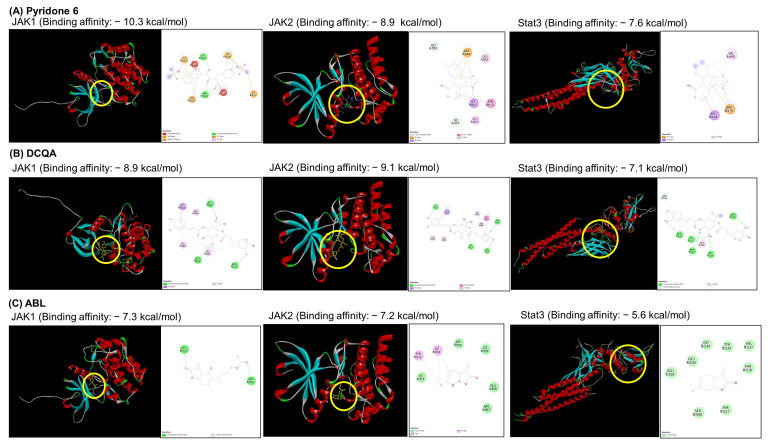
Molecular docking analysis of DCQA and ABL with JAK1, JAK2, and STAT-3. Structure-based analysis was performed to predict the binding affinity of (**A**) pyridone 6, (**B**) DCQA, and (**C**) ABL. The predicted binding sites of each molecule on JAK1, JAK2, or STAT-3 are highlighted by yellow circles.

To determine the potent interaction, docking analysis of pyridone 6, DCQA, and ABL for JAK1 and JAK2 was performed by focusing on key regions within the JAK inhibitor binding sites: the hinge region located between chains A and B of the JAK proteins. The binding affinities of pyridone 6, as a positive control, were −10.3 kcal/mol for JAK1 and − 8.9 kcal/mol for JAK2 (Figure 6A). DCQA exhibited binding affinities of −8.9 kcal/mol for JAK1 and −9.1 kcal/mol for JAK2 (Figure 6B). ABL also demonstrated the binding affinities of −7.3 kcal/mol for JAK1 and −7.2 kcal/mol for JAK2 (Figure 6C). Ligand–receptor interaction studies using each component were subsequently performed in silico. Both DCQA and ABL interact in a hydrophobic region of the ATP-binding site located in the hinge region of JAK1, adjacent to the proton acceptor active site (yellow circle in Figure 6). Both compounds also interact with the protein kinase 2 domain of JAK2, which contains a phospho-tyrosine residue for autocatalysis. Additionally, the binding potential of both compounds to the entire region of STAT3 was investigated. The binding affinity of pyridone 6 for STAT3 was −7.6 kcal/mol. DCQA displayed a binding affinity of −7.1 kcal/mol for the DNA-binding domain of STAT3, whereas ABL exhibited a low binding affinity of −5.6 kcal/mol for a region on the side of STAT3. With respect to the ligand-receptor interaction involved in STAT3 binding, DCQA might interact with the DNA-binding domain of STAT3, whereas ABL might interact with the SH2 domain of STAT3, a region crucial for phosphorylation and dimerization.

## 3. Discussion

In this study, we found that WEIF alleviated the AD pathological characteristics of DfE-induced skin inflammatory lesions by suppressing AD-like skin symptoms and decreasing total serum IgE and histamine levels and Th1/2 inflammatory mediator levels. WEIF also markedly ameliorated DfE-induced skin structure damage by restoring skin barrier protein expression. Furthermore, WEIF components suppressed the activation of JAK/STAT signaling.

DfE exposure compromises the skin barrier, inducing mast cell infiltration and a Th2 immune response, subsequently resulting in increased IgE production [18,21]. IgE binding to FcεRI on immune cells triggers the production of inflammatory mediators and regulates the release of these mediators [30]. DfE-induced production of proinflammatory cytokines and chemokines from epidermal keratinocytes and/or dermal fibroblasts exacerbates keratinocyte differentiation, leading to a defective stratum corneum [17,19], which reduces structural protein expression and impairs the structural composition of the skin, resulting in dry and itchy skin. Our results revealed that topical application of DfE elicited a significant increase in serum IgE levels, alongside remarkable skin barrier disruption and mast cell infiltration in the dorsal skin lesions of NC/Nga mice. However, we found that WEIF significantly attenuated DfE-induced AD pathological features and increased Th1/2 mediator levels in the serum of NC/Nga mice. While pathological progression to acute AD is characterized predominantly by a Th2 inflammatory profile, which is characterized by elevated Th2 mediator levels, chronic AD is characterized by a mixed Th1/Th2 response, which is characterized by increased Th1 mediator levels, such as those of IFN-γ [31,32]. However, the progression to the chronic phase of AD is further characterized by the concurrent activation of Th1 cells alongside the persistent activation of Th2 and/or Th22 cells [33]. Notably, a significant upregulation of IL-22 has been observed in the skin of chronic AD patients, which is associated with epidermal hyperplasia and the inhibition of keratinocyte terminal differentiation [34,35]. It underscores the critical role of the Th2 and Th22 cytokine axes in the pathogenesis of chronic AD. Up to now, effective AD management necessitates the balanced regulation of both Th1 and Th2 responses to address the exacerbation of T-cell immune response during AD progression [36]. In a previous study, WEIF was revealed to reduce the serum levels of Th2 cytokines (IL-4, IL-5, and IL-13) and IgE in an OVA-induced asthma model by inhibiting the JAK/STAT signaling pathway [23]. The JAK inhibitor pyridone 6 potently inhibits Th2 immunity and modestly inhibits Th1 responses, both of which are mediated through JAK/STAT signaling, which ameliorates AD-like skin inflammation [37]. Our findings demonstrated that WEIF significantly attenuated IgE levels and the levels of both Th1- and Th2-inflammatory mediators, suggesting its potential to regulate the Th1/2 imbalance during AD progression. However, further studies are needed to address the potential regulatory effect of WEIF on other immune cells, such as Th22 cells, in the context of chronic skin inflammation.

The JAK/STAT signaling pathway is pivotal in skin inflammation [12,38]. Specifically, JAK-activated STAT3 reduces the expression of structural skin proteins, such as filaggrin, leading to an increase in TEWL values in AD model mice. In addition, JAK-activated STAT6 stimulates inflammatory chemokine expression in keratinocytes, exacerbating skin inflammation [39]. However, the JAK inhibitor delgocitinib suppresses both STAT3 and STAT6 activation, which decrease the level of inflammatory mediators in human keratinocytes and improves skin barrier function by restoring skin protein expression in human skin graft models [11]. Our findings demonstrated that the WEIF components DCQA and ABL inhibit JAK1/2 and STAT3 activation in human keratinocytes. In addition, WEIF restored skin protein expression, mitigating DfE-induced skin structural damage, which lead to a decrease in TEWL levels in DfE-induced AD-like model mice. Furthermore, WEIF reduced the serum levels of IgE, histamine, and Th1/2 inflammatory mediators. A recent study revealed that the organic solvent fraction of *I. japonica* and its isolated components, such as DCQA, dose-dependently inhibited the TNF-α/IFN-γ-induced generation of RANTES, MDC, and TARC in HaCaT cells [40]. In addition, WEIF and its components, DCQA, ABL, and 6-methoxyluteolin, contribute to the inhibitory effect of WEIF on the level of the type 2 inflammatory marker periostin by suppressing JAK2-STAT3/6 activation in human bronchial epithelial cells, which ameliorates OVA-induced airway inflammation [23]. In silico studies further revealed that DCQA has a high binding affinity for the hinge region and the N- and C-terminal lobes of the JAK domain [41], which is consistent with our docking analysis of DCQA and ABL on JAK1/2. Therefore, because DCQA and ABL are major quinic acids or lactones in WEIF, WEIF might have anti-inflammatory effects on skin lesions and repair the epidermal skin barrier to alleviate AD symptoms by inhibiting the JAK/STAT signaling pathway.

Ligand-stimulated receptor activation initiates JAK activation through autophosphorylation of tyrosine residues [42]. This subsequently leads to STAT phosphorylation to induce STAT dimerization and transcriptional regulation in the nucleus. The ATP-binding pocket within the hinge region of JAK is a recognized primary target for JAK regulation [43]. Hydrogen bond formation between JAK inhibitors and amino acid residues proximal to the ATP-binding site is critical for inhibitor anchoring and subsequent abrogation of JAK signaling [44]. However, the hinge region and the area adjacent to the catalytic loop within the JAK domain are also characterized by electrostatic interactions with hydrophobic amino acid residues, which establishes a nonpolar microenvironment. JAK inhibitors frequently exploit this region by engaging with these residues via electrostatic interactions, thereby increasing their binding affinity and providing conformational flexibility to induce allosteric changes in the JAK conformation and JAK inhibition. Our findings demonstrated that the major components of WEIF, DCQA, and ABL inhibited the phosphorylation of JAK1 (tyrosine 1034/1035) and JAK2 (tyrosine 1007/1008), which are essential for kinase activation [45,46]. In addition, 3D visualization results revealed that DCQA and ABL occupy the hinge region of JAK1/2, especially JH1 protein kinase domain, which might contribute to block tyrosine phosphorylation of target proteins. Furthermore, 2D interaction analysis revealed that DCQA and ABL interact with specific amino acids in the DFG motif of JAK2 (Phe995 for DCQA and Asp994 for ABL). The DFG motif is a highly conserved sequence which functions as an “on/off’ for ATP binding and kinase activity [47]. A interaction analysis also exhibited that two components form some hydrogen bonds and several hydrophobic contacts within the JAK hinge region, suggesting that the stability of electrostatic interactions can be generated within JAK. Therefore, the combination of the hydrophobic environment and hydrogen bond formation between WEIF components and the JAK hinge region necessary for kinase function may contribute to the stable binding of WEIF components, thereby inhibiting JAK signaling. The presence of multiple phosphorylation/dephosphorylation tyrosine residues within the JAK protein indicates that complex allosteric interactions between ligand candidates and JAK domains result in varied modes and degrees of ligand-receptor association. Further investigations into the potential interactions of other WEIF components with the regulatory domains of JAK may elucidate the detailed mechanism by which WEIF inhibits JAK signaling. Moreover, evaluating the in vivo efficacy of DCQA or ABL in chronic animal models could provide valuable insight into their therapeutic potential for alleviating AD-like skin inflammation.

JAK-activated STAT3 was found to play a role in skin structure damage in AD model mice. We found that WEIF components (DCQA and ABL) significantly suppressed STAT3 activation and WEIF improved skin barrier function, as evidenced by TEWL levels, in AD model mice. However, WEIF components exhibited moderate or low binding affinity for STAT3 compared to their affinity for JAK1/2, suggesting that the observed STAT3 inhibition of WEIF components may arise from an indirect inhibitory effect on JAK, thereby affecting STAT phosphorylation rather than direct inhibition of STAT3 itself.

## 4. Materials and Methods

### 4.1. Preparation of WEIF

Dried *I. japonica* flower (Naemome Dah, Ulsan, Republic of Korea) were completely submerged in water for 24 h. The solvent was evaporated and repeatedly condensed by using a low-temperature reflux extraction method while keeping the temperature below 100 ± 2 °C. The prepared WEIF was lyophilized by using a water evaporation system equipped with a vacuum pump and a rotary evaporator. WEIF powder was dissolved in water and filtered through a syringe filter harboring 0.2 µm polytetrafluoroethylene for in vitro experiments. For in vivo experiments, the WEIF powder was dissolved in specific-pathogen-free (SPF)-grade water. We previously identified and quantified nine WEIF compounds including DCQA and ABL for mina components of WEIF using UPLC-TQ-MS/MS analysis [23].

### 4.2. Animals

All animal procedures were conducted in accordance with the ‘Guide for the Care and Use of Laboratory Animals’ (National Institutes of Health, 2013) (https://www.ncbi.nlm.nih.gov/books/NBK54050/ (accessed on 15 July 2022)). The experimental protocol was reviewed and approved by three independent reviewers on the Institutional Animal Care and Use Committee at the Korea Institute of Oriental Medicine (KIOM) (Approval No. 22-073; Approval date, 1 August 2022). Eight-week-old male NC/Nga mice (19–24 g) were purchased from Central Lab Animal, Inc. (Seoul, Republic of Korea). The health status of the mice was checked twice weekly by an animal care expert in a SPF facility of KIOM under standard animal care conditions (temperature 23 ± 2 °C, humidity 55 ± 10%, and a 12 h light–dark cycle). After a one-week acclimation period, the mice were randomly assigned to six groups (*n* = 6 per group). Standard laboratory-grade animal chow and SPF-grade water were provided ad libitum.

### 4.3. AD Induction and Dermatitis Scoring

The experimental protocol and timeline for AD induction were optimized on the basis of previous studies [9]. After the hair on the dorsal sides and ears of the mice was shaved, 200 µL of sodium dodecyl sulfate (SDS, 4%) was topically spread on the NC/Nga mice. The mice were allowed to recover for 48 h, and research-grade DfE ointment (100 mg, Biostir Inc., Kobe, Japan) was applied to the same lesions twice weekly for 21 days. Concomitant with AD induction for 7 days, the mice were orally administered three doses of WEIF (30, 100, 300 mg/kg) or dexamethasone (1 mg/kg) daily using a syringe with plastic feeding tubes for 21 days. Dermatitis scoring and ear thickness were measured by the same observer. Skin lesion severity was evaluated twice weekly using a clinical scoring system. Four clinical signs representing AD symptoms, scaling/dryness, erythema/hemorrhage, edema, or excoriation/erosion, were each assigned scores of 0 (absent), 1 (mild), 2 (moderate), or 3 (severe). A total severity score was then calculated by summing the individual scores for each of the four signs. Ear thickness was measured twice weekly at a consistent anatomical lesion on each mouse using a digital caliper (CAS©, Seoul, Republic of Korea) to reduce bias and minimize measurement variability. To evaluate the ability of the skin to retain moisture and prevent water loss, the TEWL was measured via a skin barrier light device (GPower, Seoul, Republic of Korea). TEWL was measured on the dorsal skin lesions of the mice anesthetized with pentobarbital sodium on the final day of the experiment.

### 4.4. Histological Analysis

The excised tissues were preserved in 10% formaldehyde solution (Sigma-Aldrich, Burlington, MA, USA). for fixation. After multiple washes in the appropriate buffer, the samples were embedded in paraffin for tissue cryosectioning. Hematoxylin and eosin (H&E) staining was performed to assess hyperkeratosis and epidermal hyperplasia of the skin tissue. Mast cell infiltration was evaluated using a toluidine blue (TB) staining kit (VitroVivo Biotech, Rockville, MD, USA) according to the manufacturer’s protocol. Sirius red (SR) staining for eosinophil was performed with some modification of the manufacturer’s protocol (Stemcell, Columbia, SC, Canada). The number of TB- or SR-positive cells in four random areas was determined for subsequent statistical analysis. The “number of mast cells per mm^2^ area = total number of mast cells/total area (mm^2^)” was calculated three times and used for analysis. Antibodies against filaggrin (Enzo ENZ-ABS181-0100), loricrin (Abcam, ab85679), and involucrin (Abclone, A13311) were used for immunostaining skin tissue. Morphometric analysis (immunostaining an area of the epidermis in each sample to analyze protein expression in relation to the total area of the epidermis) was performed using ImageJ 1.51 software (NIH, Bethesda, MD, USA). The stained tissue sections were digitized using a digital slide scanning system (Kfbio, Ningbo, China) in semiautomated mode for subsequent image analysis. The stained sections were examined under a microscope at 400× magnification.

### 4.5. Inflammatory Factor Analysis

Blood samples were obtained from the abdominal aorta vein of anesthetized animals 12 h after the final administration and a subsequent fasting period. Sera were separated from clotted blood by centrifugation and stored at −70 °C. Serum inflammatory factors were quantified by using antibody coated wells to capture target molecules with the LBIS Mouse IgE ELISA Kit (Fujifilm, Shibukawa, Japan), Histamine Research ELISATM (LDN, Nordhorn, Germany), or the LEGENDplex™ Mouse Proinflammatory Chemokine Panel (BioLegend) according to the protocol reported in a previous study. A spectraMax and BD LSRFortessa™ flow cytometer (BD Biosciences, San Jose, CA, USA) with BD CellQuest™ and LEGENDplex™ Software v8.0 (VigeneTech Inc., Carlisle, MA, USA) were used for detection and analysis of the immune assay results.

### 4.6. Cell Culture and Viability Assay

HaCaT human keratinocyte cells were purchased from American Type Culture Collection (Manassas, VA, USA) and cultured in Dulbecco’s Modified Eagle’s Medium containing heat-inactivated 10% FBS and 1% penicillin/streptomycin (Thermo Fisher Scientific, Waltham, MA, USA). Normal human epidermal keratinocytes (HEKs) (catalog number 00192627) (LonzaBioscience, Basel, Switzerland) were cultured in KBM-Gold Basal medium supplemented with a SingleQuots Supplement pack (LonzaBioscience). ReagentPack subculture reagents (LonzaBioscience) were used for prewashing, trypsinization, and neutralization of the cell subcultures as recommended by the manufacturer’s instructions. TNF-α and IFN-γ (10 ng/mL) were added to the medium for the activation of HEKs. Normal cell culture conditions (5% CO_2_, 37 °C) were maintained in a HeracellTM incubator (Thermo Fisher Scientific). The cell viability was determined via the quantification of generated formazan dye from a tetrazolium salt using a Cell Counting Kit-8 (CCK-8; Dojindo Molecular Technologies Inc., Rockville, MD, USA). HaCaT were treated with WEIF for 24 h. HEKs were cocultured with DCQA or ABL at concentrations of up to 20 µg/mL for 24 h. Pyridone 6 was used at a concentration of 10 nM (Sigma-Aldrich, Burlington, MA, USA). The absorbance values of the media with the added CCK-8 reagent were measured at 450 nm on a SpectraMax 340 microplate reader (Molecular Devices, San Jose, CA, USA).

### 4.7. Western Blot Analysis

The cells were extracted three times with 1 min of vortexing, followed by 5 min of incubation on ice in RIPA buffer (Intron, Seoul, Republic of Korea). The protein concentration was calculated from the relative absorbance of bovine serum albumin (BSA) as a standard using a commercial BCA assay kit (Thermo Fisher Scientific, MA, USA). Briefly, the extracted samples were incubated with a mixture of Reagent A containing bicinchoninic acid (BCA) and Reagent B containing cupric sulfate (50:1, *v*/*v*) for 30 min. The extracted proteins were denatured using SDS denaturation buffer for molecular weight-dependent separation on Mini-PROTEAN TGX precast gels (Bio-Rad, Hercules, CA, USA). The proteins on the gels were subsequently electrically transferred onto polyvinylidene fluoride membranes under semidry conditions by using the Trans-Blot Turbo Transfer system (Bio-Rad). The samples were blocked with blocking solution (Atto, Tokyo, Japan) and incubated with primary antibodies overnight. Secondary antibody incubation was performed for 1 h. Specific antibodies against phospho-STAT3 (9145), STAT3 (9139), phospho-JAK1 (74129), JAK1 (3332), phospho-JAK2 (3771), JAK2 (3230), β-actin (8457), or HRP-conjugated antibodies were purchased from Cell Signaling Technology (Boston, MA, USA). Immunoreactive signals from the antibody-incubated membranes were detected via reactions with the Super Signal West Femto Chemiluminescent Substrate (Thermo Fisher Scientific) under a densitometer camera (Bio-Rad). ImageJ (version 1.54g) was used for the quantification of chemiluminescence-induced bands.

### 4.8. Molecular Docking Analysis of DCQA and ABL

Pyridone 6 (PubChem CID: 5494425), DCQA (PubChem CID: 5281769), and ABL (PubChem CID: 75528891) structures were retrieved from PubChem (https://pubchem.ncbi.nlm.nih.gov, 29 October 2024). The human JAK1 (PDB ID: 6N7A), JAK2 (PDB ID: 3UGC), and STAT3 (PDB ID: 6TLC) structures were obtained from the Protein Data Bank (PDB) (https://www.rcsb.org, 29 October 2024). Autodock Vina and Biodiscovery Studio 2023 (version v24.1.0.23298) were used to perform molecular docking simulations of two molecules against the domain pockets of three target proteins. OpenBabel (version 3.1.1) (https://openbabel.github.io/index.html, 29 October 2024) and AutoDock (version 1.5.7) (https://mgltools.scripps.edu, 30 October 2024) software were used for the preparation and conversion of PDB files to PDBQT formats, respectively. Docking simulations were performed using the AutoDock Vina API with an exhaustiveness parameter of 75. Biodiscovery Studio 2023 was used for 2D interaction analysis between each molecule and the target protein. Biodiscovery Studio 2023 and ChimeraX (version 1.8) (https://www.rbvi.ucsf.edu/chimerax, 30 October 2024) were used for 3D visualization of the docking results.

### 4.9. Statistical Analysis

The data are presented as the means ± SEMs. One-way ANOVA followed by Tukey’s post hoc test was employed for multiple group comparisons to verify statistical significance. GraphPad Prism software was used for the analysis (Version 8.0; GraphPad Software, Inc., San Diego, CA, USA). *p* < 0.05 was considered to indicate statistical significance.

## 5. Conclusions

In this study, WEIF administration alleviated DfE-induced AD-like skin inflammation, simultaneously reduced Th immune mediator levels, and restored skin barrier protein expression. WEIF components also blocked the JAK/STAT signaling pathway in TNF-α- and IFN-γ-stimulated keratinocytes with high binding affinities for JAK proteins, which might contribute to the inhibitory effect of WEIF on DfE-induced AD-like skin inflammation. Therefore, these results provide insights into the use of WEIF or its components in the development of functional foods or therapeutic agents against AD-like skin inflammatory diseases.

## Data Availability

The data reported in this study are included in this manuscript.

## Abbreviations

ABL	1-O-acetylbritannilactone
AD	Atopic dermatitis
DCQA	1,5-dicaffeoylquinic acid
DfE	*Dermatophagoides farinae* extract
H&E	Hematoxylin & eosin
IFN-γ	Interferon-γ
IgE	Immunoglobulin E
IL-4	Interleukin-4
JAK	Janus kinase
JNK	c-Jun N-terminal kinase
MCP-1	Monocyte chemoattractant protein-1
MDC	Macrophage-derived chemokine
NC/Nga	Nishiki-nezumi Cinnamon/Nagoy
OVA	Ovalbumin
RANTES	Regulated on Activation, Normal T Cell Expressed and Secreted
SDS	Sodium dodecyl sulfate
STAT	Signal transducer and activator of transcription
TB	Toluidine blue
TEWL	Transepidermal water loss
TNF-α	Tumor necrosis factor-α
WEIF	Water extract of *I. japonica* flower
